# CG dinucleotides enhance promoter activity independent of DNA methylation

**DOI:** 10.1101/gr.241653.118

**Published:** 2019-04

**Authors:** Dominik Hartl, Arnaud R. Krebs, Ralph S. Grand, Tuncay Baubec, Luke Isbel, Christiane Wirbelauer, Lukas Burger, Dirk Schübeler

**Affiliations:** 1 Friedrich Miescher Institute for Biomedical Research, CH 4058 Basel, Switzerland;; 2 Faculty of Sciences, University of Basel, CH 4003 Basel, Switzerland;; 3 Swiss Institute of Bioinformatics, CH 4058 Basel, Switzerland

## Abstract

Most mammalian RNA polymerase II initiation events occur at CpG islands, which are rich in CpGs and devoid of DNA methylation. Despite their relevance for gene regulation, it is unknown to what extent the CpG dinucleotide itself actually contributes to promoter activity. To address this question, we determined the transcriptional activity of a large number of chromosomally integrated promoter constructs and monitored binding of transcription factors assumed to play a role in CpG island activity. This revealed that CpG density significantly improves motif-based prediction of transcription factor binding. Our experiments also show that high CpG density alone is insufficient for transcriptional activity, yet results in increased transcriptional output when combined with particular transcription factor motifs. However, this CpG contribution to promoter activity is independent of DNA methyltransferase activity. Together, this refines our understanding of mammalian promoter regulation as it shows that high CpG density within CpG islands directly contributes to an environment permissive for full transcriptional activity.

Gene regulation establishes correct spatio-temporal expression patterns essential for cellular function. Expression is controlled at multiple levels, including recognition of specific DNA sequences by transcription factors (TFs), chromatin structure, modifications of nucleosomes, and methylation of DNA. While the majority of transcription factors recognize complex motifs of several nucleotides, it is unclear whether lower complexity sequence features, such as dinucleotides, contribute independently to gene activity. CpG is the most studied dinucleotide in mammalian genomes and the site of cytosine methylation (Bird 1980; Lister and Ecker 2009; Stadler et al. 2011). In mammals, the majority of CpGs are methylated, while unmethylated CpGs are concentrated in specific regions called CpG islands (CGIs) (Bird et al. 1985). CGIs are defined as being 200 bp or longer with a G + C content of >50% and a CpG observed over expected (OE) ratio of at least 0.6 (Gardiner-Garden and Frommer 1987). Here, we will refer to the OE ratio as “normalized CpG density.” CGIs make up two thirds of all mammalian promoters, reflected in a bimodal distribution of their normalized CpG density (Fig. 1A; Mohn and Schübeler 2009). They display higher transcriptional activity than non-CGI promoters (Fig. 1B) and tend to be active across many cell types (Larsen et al. 1992). Consequently, most initiation events of RNA polymerase II in mammalian cells occur at CGI promoters.

**Figure 1.**
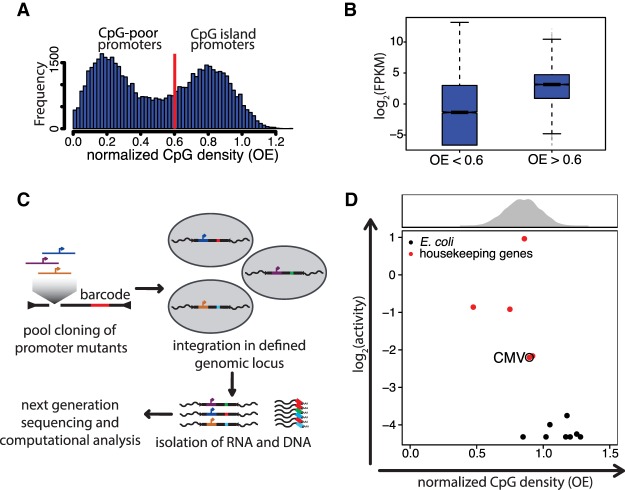
High normalized CpG density alone is not sufficient for transcriptional activity. (*A*) Histogram of CpG densities of all promoters in the mouse genome (400 bp upstream to 200 bp downstream from TSS). Normalized CpG density is distributed in a bimodal fashion. CpG density was calculated as the observed to expected ratio (OE = [number of CpGs/{number of Cs × number of Gs}] × length of the region in nucleotides). The red line indicates the threshold in OE used in the standard definition of CpG islands (Gardiner-Garden and Frommer 1987). (*B*) Box plot displaying transcriptional activity of CpG-poor (OE < 0.6) and CpG-rich (OE > 0.6) promoters, as measured by RNA sequencing in embryonic stem cells (data from Domcke et al. 2015). (FPKM) Fragments per kilobase per million mapped reads. (*C*) Schematic representation of the procedure used to perform parallel reporter assays in a defined genomic locus. Promoter mutants are batch-cloned in front of *GFP* as a spacer sequence and a unique barcode. The expression cassette is flanked by *loxP* sites that allow integration into the beta-globin locus of the embryonic stem cell line, replacing a selection cassette. After selection for cells containing the reporter construct, DNA and RNA are isolated and the latter reverse-transcribed. Barcodes are PCR-amplified and sequenced. Normalization of RNA barcode frequency to DNA barcode frequency results in relative expression levels between constructs. (*D*) CpG density versus transcriptional activity of sequences from the *Escherichia coli* genome (black dots) and active housekeeping genes (HKG, red dots) inserted into embryonic stem cells. The CMV promoter is indicated for reference as an example of a lowly active promoter. The histogram *above* the scatter plot depicts the normalized CpG density distribution of CGI promoters.

Why CpG density is increased in CGIs remains unclear, as well as whether the CpG dinucleotide plays a role in transcriptional and/or epigenetic regulation. One explanation portrays CGIs as a footprint of evolution due to lower mutation rates of unmethylated CpGs (Bird 1980). In support of this, unmethylated cytosines deaminate to uracil (Barnes and Lindahl 2004), an improper DNA base that is efficiently repaired. In contrast, methylated cytosines deaminate to thymidine, a proper genomic base that is less efficiently repaired, resulting in a higher C→T mutation rate. While this model is supported by comparative genomics (Cohen et al. 2011) and could explain the presence of CGIs, it does not address a regulatory function of CpG dinucleotides. Some CpGs operate as part of larger motifs and thus serve to recruit TFs. Furthermore, TF binding can keep CpGs unmethylated, as suggested for SP1 (Brandeis et al. 1994; Macleod et al. 1994), while methylated CpGs can repel or even enhance binding (Domcke et al. 2015; Kribelbauer et al. 2017; Yin et al. 2017). There is limited evidence for evolutionary selection of CpGs to reside in defined positions (Cohen et al. 2011), arguing that only a minority of CpGs are part of larger motifs. This suggests a neutral evolutionary regime in which CpGs come and go in a mostly random fashion within CGIs. Importantly, the latter does not exclude a functional contribution. CpG density alone can protect DNA from methylation (Lienert et al. 2011; Krebs et al. 2014; Wachter et al. 2014; Long et al. 2016), and CpG dinucleotides have been suggested to further act as a signaling module (Bird 2011). A possible mechanism involves ZF-CxxC domain proteins, which bind unmethylated CpGs (Long et al. 2013). Several chromatin modifying enzymes contain CxxC domains, and some are proposed to counteract methyltransferase activity (Ooi et al. 2007; Cedar and Bergman 2009).

Taken together, CpGs could have a general effect on promoter activity that is distinct from their occurrence as a part of complex TF motifs. Distinguishing these scenarios is not trivial. It requires knowledge of TF binding within CGIs and testing the contribution of CpGs to promoter activity. While the former can be addressed using ChIP-seq, the latter requires a reporter assay that quantifies transcriptional output as a function of sequence mutations. Due to the high frequency of the CpG dinucleotide, this requires many mutations and measuring many variants. Thus far, most high-throughput transcriptional reporter assays in higher eukaryotes used transient transfection (Patwardhan et al. 2009, 2012; Kwasnieski et al. 2012; Melnikov et al. 2012; Mogno et al. 2013; White et al. 2013; Shen et al. 2015). Chromosome integration is, however, desirable given the reported differences in transcriptional activity from episomes or varied chromosomal location (Inoue et al. 2017).

Here, we investigate the contribution of CpGs to transcriptional activity and binding of transcription factors to their motifs in CGIs. We contrast hundreds of mutant sequences after inserting them into the same genomic site in mouse embryonic stem cells (ESCs). The resulting loss- and gain-of-function experiments reveal that CpGs contribute to transcriptional output independent of DNA methylation.

## Results

### Parallel reporter assay at a defined chromosomal site

Investigating CpG function necessitates an approach that systematically compares different sequences in parallel and in the context of chromosomal DNA. This requires a sequencing strategy that links RNA molecules (i.e., expression counts) to upstream regulatory regions that are not part of the transcript. Toward this goal, we designed a parallel reporter assay called TrAC-seq (Transcriptional Activity in Chromatin). With TrAC-seq, promoter sequences are cloned in a pooled format and inserted into a defined genomic locus. The resulting transcripts are sequenced and assigned to their specific promoters using barcodes (BCs) (Fig. 1C). BC frequencies are quantified by isolating RNA and DNA and sequencing the BCs from both. Resulting barcode frequencies in the RNA are then normalized to the frequency of the actual template using the representation of the same BC in the DNA of the cell population (Fig. 1C; Methods).

To control for the contribution of chromatin and the local genetic environment, we integrated the library of promoter-barcode constructs into the beta-globin locus in ESCs using recombinase-mediated cassette exchange (RMCE) (Lienert et al. 2011; Krebs et al. 2014). This region is transcriptionally silent outside the erythroid lineage (Fromm and Bulge 2009).

The mean signal of all BCs corresponding to one promoter allowed us to reproducibly quantify the relative activity of promoters within a pool (Supplemental Figs. S1A,B, S4A,B, S4E–G, S5A,B), enabling the measurement of up to ∼3100 promoter-BC constructs within a single experiment. In total, we tested more than 10,000 promoter-BC constructs representing ∼270 unique promoter sequences.

### High density of CpGs alone does not confer CGI activity

Normalized CpG density correlates well with transcriptional activity of endogenous promoters (Fig. 1B), but whether or not this is a direct consequence of CpG density remains unclear. High CpG density coincides with features of transcriptionally permissive chromatin, such as trimethylation at lysine 4 on histone H3 (H3K4me3) and a lack of DNA methylation at endogenous and artificial sequences (Lienert et al. 2011; Krebs et al. 2014; Wachter et al. 2014). To assess if high CpG density alone is sufficient for transcriptional activity, we tested the activity of sequences from a prokaryotic genome (*Escherichia coli*). These have a CpG density comparable to CGIs but have not evolved binding sites for eukaryotic TFs. Combined with a minimal promoter, their activity, however, is barely detectable by TrAC-seq compared to a selection of active housekeeping gene promoters (Fig. 1D), showing that high CpG density is insufficient for transcriptional activity on chromatin. Even if insufficient for activity, CpGs could nevertheless contribute to CGI activity. To test this requires mutating CpGs and monitoring the effect on activity. A careful design of such mutations is needed to distinguish between CpGs that are part of complex TF motifs (motif-CpGs) and those that are not (non-motif-CpGs). Since binding motifs are generally poor predictors of actual TF binding due to many unoccupied motif occurrences (Biggin 2011), we determined actual binding of selected TFs using ChIP-seq.

### TF motifs within CpG islands are preferentially bound

**Figure 2.**
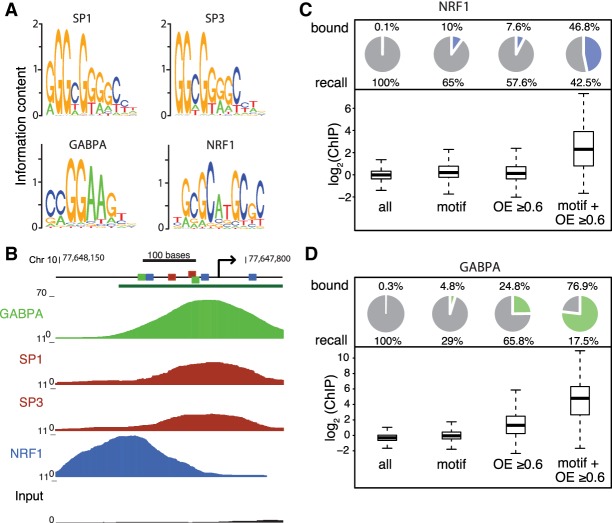
Bound TF motifs are enriched in CpG islands. (*A*) Position weight matrices of SP1, SP3, GABPA, and NRF1 as inferred from the respective ChIP-seq peaks. (*B*) Browser screenshot of SP1, SP3, GABPA, and NRF1 ChIP-seq data sets at the *Pwp2* promoter. Predicted TF motifs for the respective factors are highlighted as colored squares; the green bar indicates a CpG island. (*C*) High CpG density and TF motif occurrence combined result in the largest enrichment of bound TF motifs. Pie charts show, for different subsets, the percentage of NRF1-bound genomic windows (600-nt tiling windows, log_2_ enrichment over input >2.5), with the percentage indicated above the pie chart (bound); (recall) percentage of all bound genomic windows that are part of each subset. Corresponding box plots of log_2_ ChIP enrichments are shown *below* the pie charts. (All) All windows, (motif) windows containing a motif that has a log-odds score ≥ 12 (log_2_ scale), (OE ≥ 0.6) windows with an OE ≥ 0.6, (motif + OE ≥ 0.6) windows with both a motif with a log-odds score ≥ 12 and an OE ≥ 0.6. (*D*) Same as in *C* for the first replicate of GABPA.

We profiled four TFs with CpGs in their canonical motifs and relatively broad expression pattern (Fig. 2A; Supplemental Fig. S2A–D; data from Shen et al. 2012). Among these, SP1 and SP3 were implicated in regulating a CGI promoter (Brandeis et al. 1994; Macleod et al. 1994), while we previously profiled NRF1, revealing its binding inhibition by DNA methylation (Domcke et al. 2015). We profiled SP1, SP3, and GABPA in mouse embryonic stem cells using the “Rambio” approach (Supplemental Fig. S2E–G; Baubec et al. 2013), yielding reproducible ChIP-seq data for all factors (Supplemental Fig. S2H). Binding was indistinguishable between SP1 and SP3, which recognize similar low complexity motifs and displayed comparably low enrichments (Supplemental Fig. S2I). This limited an in-depth analysis of binding sites but allowed the classification of promoters as bound or unbound (Fig. 2B). Local enrichments were considerably higher for NRF1 and GABPA, enabling a detailed analysis (Fig. 2C,D; Supplemental Fig. S3A).

As expected, presence of motifs of at least intermediate motif score are a poor predictor of binding for these two factors, since only 10% and ∼5% of all genomic windows with NRF1 and GABPA motifs are bound, respectively (Fig. 2C,D; Supplemental Fig. S3A). To test how CpG frequency relates to binding, we contrasted CpG density with TF binding independent of motif. For both factors, binding is more prevalent in windows with higher CpG density (OE ≥ 0.6) (Fig. 2C,D). For GABPA, enrichments in high CpG density windows are larger than in those that harbor a motif of at least intermediate score (Fig. 2D; Supplemental Fig. S3A). Windows with both high CpG density and motif occurrence are bound at high frequency, with NRF1 occupying ∼45% and GABPA ∼70% of these windows (Fig. 2C,D; Supplemental Fig. S3A). They account for ∼40% of all binding events for NRF1 and ∼15%–20% for GABPA.

To move beyond this binarized comparison, we explored the predictive power of CpG and motif over a continuous range. This revealed that binding increases with CpG density and starts to diminish around the CGI threshold (Supplemental Fig. S3B,C). For GABPA, CpG density predicts binding better than motif alone, which is not the case for NRF1 (Supplemental Fig. S3B,C). Combining motif score and regional CpG density in an additive model using logistic regression improves the predictive power over individual measures (Supplemental Fig. S3B,C; Methods). If only windows with OE ≥ 0.6 are considered, almost all windows with top-scoring motifs are bound (roughly 90% for GABPA and 80% for NRF1) (Supplemental Fig. S3B,C). Taken together, this suggests that increasing CpG density does not simply enrich for bound motifs because the motifs themselves contain a CpG, but that CpG density itself contributes to binding, which, in turn, might affect transcriptional output of CGI promoters.

### CpG density contributes to CGI activity

**Figure 3.**
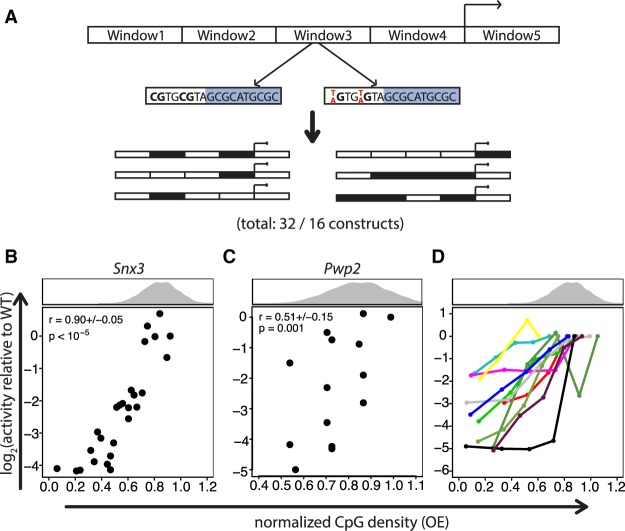
Normalized CpG density correlates with transcriptional activity. (*A*) Mutation strategy. Promoters were mutated in windows, in which all Cs within CpGs that were not a part of a complex motif of TFs with a ChIP-seq peak at the promoter were mutated to Ts (for *Snx3*) or As (for *Pwp2*). WT and mutant windows were assembled in all possible combinations and assayed for transcriptional activity. The windows in the *Snx3* promoter had 5, 10, 6, 9, and 12 CpGs, respectively, and ranged from 50–120 bp in size. For *Pwp2,* windows of 70–150 bp with 8, 7, 7, and 7 CpGs, respectively, were mutated. The numbers 32 and 16 indicate the number of constructs for *Snx3* and *Pwp2*, respectively, the large majority of which led to a transcriptional read-out. (*B*) Presence of CpGs positively correlates with transcriptional activity. Scatter plot of normalized CpG density versus transcriptional activity of *Snx3* promoter mutants. The histogram *above* the scatter plot depicts the normalized CpG density distribution within promoters overlapping CGIs. The average Spearman's correlation coefficient of all three replicates (±1 SD) and its significance is indicated in the *upper left* part of the plot. *P*-values were determined based on an approximate permutation test (see Methods). (*C*) Same as in *B* for the *Pwp2* promoter. Due to low coverage of BCs for this promoter series, in this case we adjusted the threshold on the minimal number of required BCs per promoter mutant to 1. (*D*) Positive correlation of normalized CpG density with transcriptional activity is a general feature in promoter mutant libraries. Scatter plot showing normalized CpG density versus transcriptional activity in the reporter assay for 11 promoters. Mutant promoters were generated by random mutation of Cs to As within CpGs if they were not part of complex motifs of TFs that have a ChIP-seq peak at the promoter. Different numbers of CpGs were mutated to generate five different normalized CpG densities per promoter.

To test the contribution of CpGs to transcription, we measured 78 broadly active CGI promoters with TrAC-seq (Supplemental Fig. S4A,B), which displayed variable expression (Supplemental Fig. S4C,D). We focused on two promoters with high activity (*Snx3* [∼400 bp] and *Pwp2* [∼460 bp]) and systematically mutated their CpGs. To reduce the likelihood of changing motif-CpGs, we used the binding data described above to identify bound motifs. We divided each promoter into four (*Pwp2*) or five (*Snx3*) regions and generated all possible combinations of regions with either wild-type (WT) sequence or regions in which all CpGs outside of bound TF binding sites of SP1, SP3, GABPA, and NRF1 were mutated (Fig. 3A). TrAC-seq of this library revealed for both promoters that activity decreases with decreased CpG density (Fig. 3B,C), suggesting that non-motif-CpGs contribute to transcriptional activity.

To ask if this observation can be generalized, we mutated 11 additional CGI promoters spanning a range of normalized CpG density and transcriptional activity (Fig. 3D; Supplemental Fig. S4H). This time, CpGs were randomly chosen to generate elements with 100%, 75%, 50%, 25%, and 0% of non-motif-CpGs. Here, we included an additional 17 published ChIP-seq data sets to define non-motif-CpGs (Supplemental Table S3). Moreover, CpGs to be mutated were randomly selected for each CpG density to ensure that effects are not due to absence of the same set of mutated CpGs of a particular promoter. This more comprehensive set of promoters shows a similar response; removing non-motif-CpGs lowers the transcriptional activity in most cases (Fig. 3D).

While these experiments argue for a general contribution of non-motif-CpGs to CGI transcriptional activity, we cannot exclude that some of the effect may be due to mutations of CpGs that reside within nonverified TF motifs.

An indication of this may be the higher spread of activities at similar CpG densities for the *Pwp2* promoter compared to *Snx3* (Fig. 3B,C). Since we mutate windows of CpGs in different combinations (Fig. 3A–C) or individual CpGs in a random fashion (Fig. 3D), this seems unlikely. Nonetheless, we applied a more controlled mutation approach to the *Pwp2* promoter to test this possibility.

### Dissecting CpGs from TF motifs

**Figure 4.**
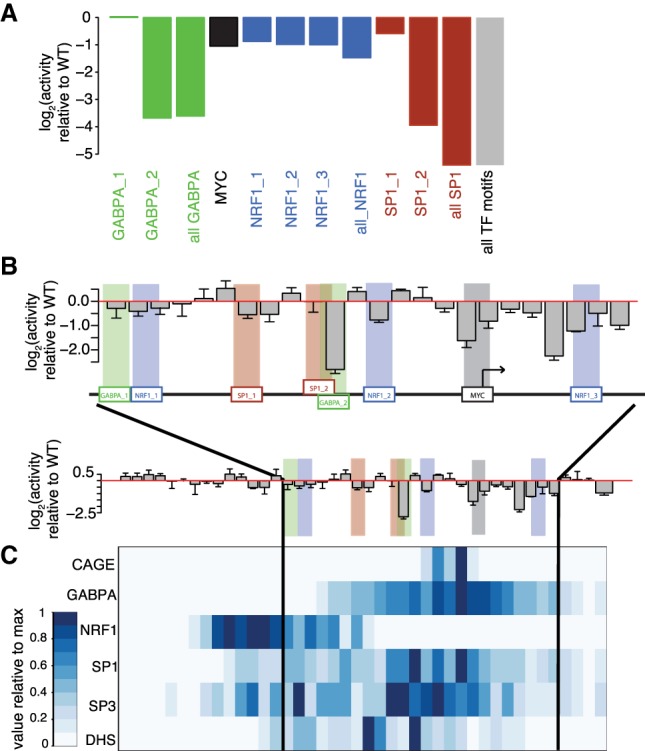
Characterization of the *Pwp2* promoter. (*A*) Mutation of specific TF motifs leads to decreased transcriptional activity. Bar plots showing log_2_ activity relative to WT constructs with single TF motif mutations or mutations of all motifs of each TF. Due to low coverage of BCs per promoter, we adjusted the threshold on the minimal number of required BCs per promoter mutant to 1. (*B*) Mutation of 10-bp windows reveals highly variable effects on promoter activity. Bar plots showing transcriptional activity relative to the WT construct of promoters with mutated windows near the TSS (*top*), where most changes are seen, compared to the entire promoter (*bottom*). Tiling 10-bp windows were mutated to a random CpG-free sequence to assess the contribution of each window to transcriptional activity. Error bars show ±1 SD of three replicates. A schematic view of a region of the *Pwp2* promoter that contains TF motifs (shown in colored boxes) and the TSS (indicated by arrow) is shown *between* the bar plots. (*C*) TF binding partially overlaps with regions important for transcriptional activity. Heat map displaying reads per 10-bp window for mRNA 5′ ends (CAGE), GABPA, NRF1, SP1, SP3 ChIP-seq, and DNase I hypersensitivity mapping at the endogenous *Pwp2* promoter (DHS). Scale is equal to promoter representation at the *bottom* of panel *B*.

To delineate functionally relevant motifs in the *Pwp2* promoter, we first located bound and unbound motifs of GABPA, SP1, MYC, and NRF1 using ChIP-seq data and measured transcriptional activity in constructs with mutated motifs (Fig. 4A). Upon mutation of GABPA and SP1, activity strongly decreases for one out of two motifs. For both factors, ChIP-seq signal is highest closer to the motif causing decreased activity when mutated. Especially for closely spaced SP1 motifs, it is unclear whether the resolution of ChIP-seq is sufficient to assign binding. For MYC and NRF1 motifs, activity decreases weakly upon mutation irrespective of binding. Thus, although binding does not predict activity, masking of CpGs that lead to large changes in activity when mutated appears a reasonable rationale to enrich for non-motif-CpGs. Since mutating all CpGs is not feasible, we divided the *Pwp2* promoter into tiling windows of 10 bp and generated all possible constructs with one of the windows replaced by a random 10-bp CpG-free sequence. Measuring the resulting 42 mutants after genomic insertion revealed variable effects on promoter activity. About half of the mutated 10-bp windows did not have a clear effect on expression (Fig. 4B). Some windows, however, showed a clear reduction in activity when mutated. While there is no general correspondence between predicted motifs in windows and reduced activity, the replacement with the strongest effect overlaps one GABPA motif (GABPA_2) and partially one SP1 motif (SP1_2), both of which reduce activity when mutated individually (Fig. 4A). The window with the second strongest reduction does not contain a predicted motif indicating other binding events. Motifs with moderate effects on activity when mutated individually tend to lie in windows with moderate decreases in activity. Comparison of SP1/SP3 ChIP-seq signal at the endogenous *Pwp2* promoter and transcriptional activity of mutants shows that ChIP-seq, as expected, lacks the spatial resolution to correctly discriminate binding between closely spaced motifs and, in turn, predict the effect on activity (Fig. 4B,C). If motifs are located at a larger distance, like for GABPA, ChIP-seq can indeed be sufficient to predict if specific motifs are bound (Fig. 4B,C).

Additionally, activity also decreases when mutating regions downstream from the dominant initiation site of the endogenous promoter as measured by CAGE (cap analysis by gene expression) (Fig. 4B; The FANTOM Consortium and the RIKEN PMI and CLST [DGT] 2014), suggesting that these regions contribute to initiation.

Having characterized the regulatory function of the *Pwp2* promoter at 10-bp resolution enabled us to define regions critical for transcriptional activity due to TF binding and non-motif-CpGs for further mutational analysis.

### Mutation of CpGs within regions not critical for transcriptional activity

**Figure 5.**
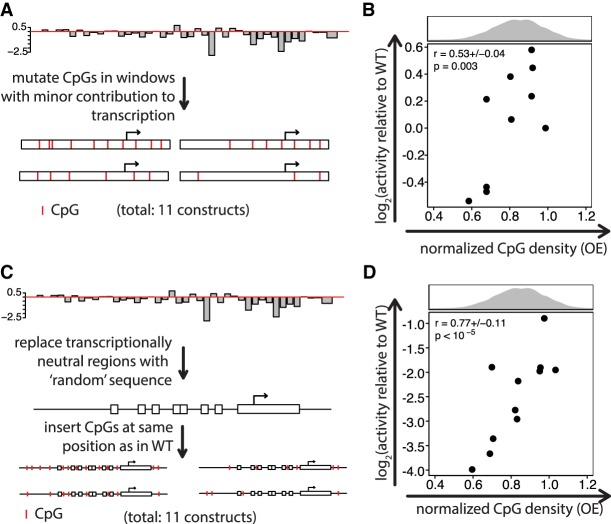
CpGs outside of TF motifs contribute to transcriptional activity of CGIs. (*A*) Mutation strategy. Cs in CpGs were mutated to As within 10-bp windows that showed small or no effect on activity when mutated. CpGs were mutated in random combinations within mutant promoter constructs. (*B*) CpGs outside of regions with a strong effect on transcriptional activity contribute to transcriptional activity. Scatter plot of normalized CpG density versus transcriptional activity relative to WT *Pwp2* for promoter mutants. Normalized CpG density correlates significantly with transcriptional activity. The average Spearman's correlation coefficient for all three replicates ±1 SD) and its significance is indicated in the *upper left* part of the scatter plot. *P*-values were determined based on an approximate permutation test (see Methods). (*C*) Mutation strategy to generate an artificial sequence context and strategy for adding back CpGs. We first determined all 10-bp sequence blocks of the *Pwp2* promoter with no effect or a weak effect on their activity when mutated and replaced them with random CpG-free sequences to retain correct spacing. Subsequently, different numbers of CpGs were re-introduced into the random CpG-free sequences at the same spatial locations as in the WT *Pwp2* promoter. (*D*) Normalized CpG density itself contributes to CGI activity. Scatter plot of normalized CpG density versus transcriptional activity relative to the activity of WT *Pwp2* for constructs in *C*. Normalized CpG density positively correlates with transcriptional activity. The average Spearman's correlation coefficient of all three replicates (±1 SD) and its significance is indicated in the *upper left* part of the scatter plot. *P*-values were calculated as in *B*.

Next, we mutated only CpGs of the *Pwp2* promoter lying in regions with minor or no effect on activity. We randomly selected subsets of these CpGs, generated 11 mutants with different CpG densities (Fig. 5A), and tested their activity. This revealed that removing non-motif-CpGs decreases transcriptional activity, resulting in a general positive correlation between CpG density and activity. More specifically, a decrease in activity relative to WT can be observed at CpG densities around 0.6–0.7 OE (Fig. 5B), where 0.7 corresponds to 12 CpGs mutated out of 35. Here, the construct with highest CpG density has up to ∼50% lower activity than other constructs with slightly lower CpG density (Fig. 5B). While this appears to be the case for a small number of additional tested constructs (Fig. 3B,D), further experiments would be required to clarify if this is a general effect.

Taken together, these findings again suggest that non-motif-CpGs contribute to transcriptional output. Finally, we wanted to test this model in a gain-of-function assay.

### Increasing CpGs within an artificial sequence context enhances promoter activity

To directly examine if CpGs alone contribute to transcriptional activity, we increased CpG density in a random sequence context. More specifically, we exchanged regions of the *Pwp2* promoter with no role or a minimal role in transcriptional activity with CpG-free sequences (Fig. 5C). This replaced ∼60% of the sequence, decreasing CpG density from ∼1 to ∼0.6 OE ratio. Using this as our baseline sequence, we reintroduced CpGs into the CpG-free regions at the same positions as in WT but in random combinations and quantified the effect on transcriptional activity. This revealed that increasing CpG density alone resulted in a gradual activity increase (Fig. 5D). We were able to regain up to ∼26% of WT promoter activity when reintroducing an equal number of CpGs as in WT (*n* = 20) and increased the activity as high as ∼54% of WT by adding more CpGs (Fig. 5D). As in Figure 5B, the construct with the highest CpG density does not have the highest activity (Fig. 5D).

When these findings are taken together, we conclude that increasing CpG density enhances promoter activity, providing further support for CpG density contributing to CGI activity.

### DNA methyltransferase activity does not account for transcriptional effect

Since high CpG density antagonizes DNA methylation of chromosomally inserted sequences (Lienert et al. 2011; Krebs et al. 2014), reducing CpGs could lead to DNA methylation, which, in turn, might account for activity reduction.

**Figure 6.**
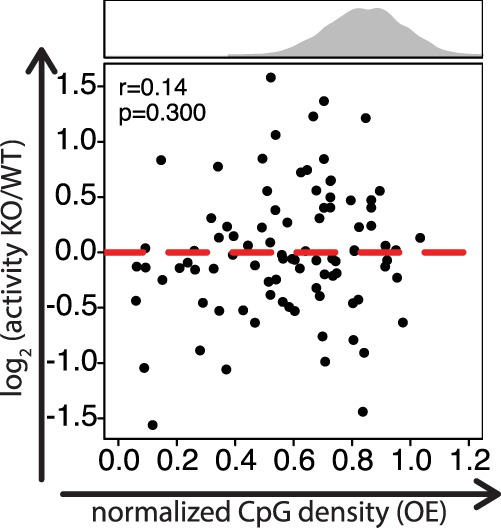
DNA methylation does not affect transcriptional activity of mutant promoters with low normalized CpG density. Scatter plot showing normalized CpG density versus log_2_ activity fold change of WT compared to *Dnmt* TKO cells. No significant dependence is observed. The Spearman's correlation coefficient and its significance are indicated in the *upper left* part of the figure. *P*-values were determined based on an exact permutation test (see Methods).

To test this, we repeated selected activity measures (Figs. 3B–D, 5B,D) in cells that lack DNA methyltransferase activity. We generated a *Dnmt1*, *Dnmt3a*, and *Dnmt3b* triple-knockout (TKO) from our parental line and performed the same genomic integration (Supplemental Fig. S5C,D). We then tested CpG density promoter mutants and compared their activity to the wild-type parental cells. This revealed that CpG contribution to promoter activity is independent of DNA methyltransferases (Fig. 6).

Next, we measured the actual DNA methylation of several mutant CGI promoters (constructs in Fig. 3D) by bisulfite sequencing of individual clones. This revealed that sequences with low CpG density indeed show an increase in DNA methylation (Supplemental Fig. S5E), in line with our previous findings that CpGs indeed protect against DNA methylation. These CpG-poor promoters (i.e., non-CGIs) show very low to no transcriptional activity in both wild-type and DNA methyltransferase TKO cells. We conclude that within the tested constructs, de novo methylation occurs at already inactive CpG-poor promoters but does not account for differential expression of CpG-rich and CpG-poor promoters.

## Discussion

By combining genome-wide profiling of TFs with high-throughput genomic insertion of promoter mutants, this study shows that CpGs are not sufficient but necessary for full activity of CGIs.

In order to obtain such comprehensive data, the development of a parallel reporter assay proved essential. Previous studies described substantial differences in transcriptional activity of constructs depending on chromosomal/episomal context (Inoue et al. 2017) or genomic location (Akhtar et al. 2013). In the current study, we introduce TrAC-seq, which enabled reproducible and sensitive measurements from constructs after insertion into the same genomic locus. As a result, only one construct is tested per cell, yet multiple measurements were obtained for each tested sequence within the cell population. This sensitivity allowed quantification of subtle changes in transcriptional activity. This assay can also be utilized to explore other sequence features of promoters or enhancers. Combined with two other approaches to measure in parallel at the same genomic site which were reported following submission of our work (Maricque et al. 2018; Weingarten-Gabbay et al. 2019), this largely extends the toolset to study *cis*-acting sequences.

Here, TrAC-seq allowed us to iterate sufficient mutant constructs to show unequivocally that CpG dinucleotides contribute to activity regardless of being in a complex motif. This is evident when removing CpGs in regions that are not critical for activity but also when adding CpGs in an otherwise random sequence context. This provides functional evidence to correlative observations linking high CpG density to active chromatin marks and high expression (Guenther et al. 2007; Weber et al. 2007; Thomson et al. 2010; Deaton and Bird 2011; Fenouil et al. 2012; van Arendsbergen et al. 2016), as well as the findings that CpG-dense sequences are free of DNA methylation when inserted into the genome (Lienert et al. 2011; Krebs et al. 2014).

A previous study suggested that the open chromatin structure of CGIs depends on high G and C content as well as high CpG density (Wachter et al. 2014). Since we focused on CpGs in our study, we cannot formally exclude a contribution of G and C content to transcriptional activity. However, we observe clear transcriptional effects upon mutations of CpGs that cause only small changes of G + C content (Supplemental Fig. S4I), strongly arguing that primarily CpG density increases activity.

CpGs might support transcriptional activity indirectly, by increasing DNA accessibility and thereby facilitating TF binding. In agreement with this model, accessibility of genomic regions correlates with CpG density (Supplemental Fig. S3D). This is consistent with studies showing that CpG-rich artificial sequences display marks of open chromatin (Lienert et al. 2011; Krebs et al. 2014; Wachter et al. 2014) and the fact that the TFs tested in our study preferentially bind their motif when located in CpG-rich regions. This relationship raises the question of whether or not accessibility decreases upon CpG depletion in the tested promoter mutants. While of interest, current accessibility techniques employ nucleases or transposases and thus are not suitable for studying libraries that are heterogeneous between cells and would require locus-specific PCR for detection.

While it remains open how CpGs mediate increased activity, recruitment of binders of unmethylated CpGs such as ZF-CxxC domain-containing proteins is one option. These are present at CGIs and correlate with their accessible chromatin environment (Blackledge et al. 2010; Clouaire et al. 2012, 2014; Boulard et al. 2015). They include CFP1, which is part of the H3K4 methyltransferase complexes SETD1A and SETD1B (Clouaire et al. 2012), and KDM2A, which removes H3K36me2 (Blackledge et al. 2010), a chromatin mark that interferes with transcriptional initiation (Strahl et al. 2002; Carrozza et al. 2005; Youdell et al. 2008; Li et al. 2009). In addition, the ZF-CxxC domain-containing protein FBXL19 has been linked to CDK-Mediator complex recruitment, representing another potential pathway (Dimitrova et al. 2018). Since the mouse and human genomes encode at least 12 different ZF-CxxC domain-containing proteins, it is challenging to functionally test their role (Long et al. 2013; Xu et al. 2018). Moreover, additional proteins can recognize unmethylated CpGs such as the zinc finger and BTB domain containing protein 2 (ZBTB2) (Karemaker and Vermeulen 2018).

Mutation to very low CpG densities increases DNA methylation, in line with previous transgenic experiments, where DNA methylation occurs most frequently at low CpG densities and rarely at CpG-rich DNA sequences (Lienert et al. 2011; Krebs et al. 2014). However, removal of DNA methyltransferase activity does not lead to specific up-regulation of the tested constructs, indicating that DNA methylation is not responsible for decreased activity upon CpG depletion in this setting.

We previously argued that DNA methylation is generally repressive at high CpG density (Schübeler 2015), while at CpG-poor sequences a repressive effect likely requires DNA methylation-sensitivity of TFs as shown for NRF1 (Domcke et al. 2015). A protective function of high CpG density against DNA methylation is a potential explanation why high CpG density together with motif occurrence is such a good predictor for TF binding for GABPA and NRF1. Protection from DNA methylation at CGIs could again be mediated by ZF-CxxC domain-containing proteins like KDM2B. Its deletion results in slow yet cumulating DNA methylation at inactive CGIs in stem cells (Boulard et al. 2015).

The transcriptional effects of mutating CpGs were rather uniform, regardless if positioned upstream or within the site of transcriptional initiation. Together, this supports a model where most CpGs within CGIs have no local function, while the overall CpG density in the promoter nevertheless enhances transcriptional activity.

How does this finding relate to models of the evolutionary origin of CGIs? Previous analysis indicated that the high CpG content in CGIs can be explained by a neutral effect of slow deamination associated with the lack of methylation, revealing no evidence for purifying selection on CpGs (Cohen et al. 2011). This is fully compatible with our observation that overall CpG density is important, rather than individual positions. It is further tempting to speculate that methylation of CpGs in the context of CGIs would interfere with their enhancing activity, which might in part account for the transcriptional repression of methylated CGIs.

The link reported here between CpG density and transcriptional activity at CGI promoters exposes a function of dinucleotide frequencies outside of complex TF motifs. Given the different structure of CpG-poor promoters and enhancers, other low-complexity motifs or resulting sequence features such as DNA shape (Zhou et al. 2015) might also operate as an additional means of fine-tuning regulation.

Taken as a whole, our study underlines the importance and complexity of sequence context beyond complex TF motifs for transcriptional activity and provides an experimental framework for rigorous testing of putative regulatory roles.

## Methods

### Cell culture

Mouse ES cells were cultured as described (Lienert et al. 2011). For detailed descriptions of cell lines, see Supplemental Methods.

### Reporter assay

#### Generation of a barcoded reporter vector

A cassette containing a *loxP* site, multiple cloning site, poly(A) signal, and another *loxP* site was synthetized and cloned into a plasmid backbone containing ampicillin resistance (Lienert et al. 2011). Barcodes were generated by annealing CGCCGAANNNNWNNNNWNNNNNAGCTCGG and TCGACCGAGCTNNNNNWNNNNWNNNNTTCGGCGCATG. The vector was cut using SphI and SalI and ligated with the annealed barcodes using T4. The ligation was precipitated, and 100 ng were transformed into MegaX DH10BT1^R^ Electrocomp Cells (Thermo Fisher Scientific). A dilution of 1:10,000 was distributed on a LB agar plate containing 50 mg/L ampicillin to estimate transformation efficiency. The rest was incubated in 50 mL LB containing 50 mg/L ampicillin shaking at 300 rpm at 37°C overnight. Plasmids were isolated using a Qiagen Plasmid Midi kit.

#### Library cloning and RMCE

Promoter libraries were cloned into the expression vector using ClaI and NheI restriction enzymes, aiming for at least 10× more colonies than unique promoters. To link barcodes and promoters, the promoter-BC fragment was amplified with primer DH.P39 (Supplemental Table S1) and one of the indexing primers containing the Illumina flow cell annealing sequences using Phusion Hot Start II polymerase (Thermo Fisher Scientific). PCR products were purified using AmPure XP beads (Beckman Coulter, #A63880) and directly sequenced using MiSeq 500- or 600-cycle kits (Illumina). The vector was cut with SphI and PacI or NheI, and a sequence containing a CpG-free *eGFP* and the annealing sequence for primer DH.P6 (Supplemental Table S1) was inserted. For an alternative construct, the insert contained a 31-bp minimal promoter in front of *eGFP*. RMCE was performed as described (Krebs et al. 2014).

#### RNA/DNA isolation and preparation for next-generation sequencing

RNA was isolated from cell lines containing the expression libraries with a Qiagen RNeasy Mini kit with on-column DNase digestion and reverse-transcribed using Takara PrimeScript RT Reagent kit (#RR047A). For DNA isolation, cell pellets were suspended in Bradley Buffer, 6 μL RNase A (10 mg/mL) was added, and samples were incubated (1 h at 37°C). Subsequently, 30 μL Proteinase K (1 mg/mL) was added, and samples were incubated at 50°C overnight. Then, DNA was extracted using Phenol:Chloroform. DNA and cDNA barcodes were amplified with KAPA HiFi HotStart using primer DH.P6 and indexing primer (Supplemental Table S1). PCR products were purified using AmPure XP beads (Beckman Coulter, #A63880) and sequenced using a 50-cycle kit on HiSeq 2500.

#### Promoter methylation analysis

Cells containing integrated mutant CGI promoter libraries were plated at low density, 96 clones picked and expanded to a minimum of 20,000 cells before lysing with Bradley Buffer (10 mM Tris-HCl (pH 7.5), 10 mM EDTA (pH 8.0), 0.5% SDS, 10 mM NaCl), and DNA extracted. DNA was bisulfite-converted using a Zymo lightning conversion kit (D5046) and cleaned up using MagBeads (Zymo). Converted DNA was amplified with primers RSG353 and RSG354 (Supplemental Table S1). Amplified DNA was purified with Ampure beads, and sequencing libraries were prepared using the Illumina NEBNext ChIP-seq library prep kit with 96 dual indexing and sequenced on a MiSeq (600 cycles).

### Generation of biotin-tagged TF cell lines

Biotin-tagged TF cell lines were generated as described (Baubec et al. 2013). Bio-GABPA was expressed under control of a CAG as well as a CMV promoter, while Bio-SP1 and Bio-SP3 were expressed using Tet-inducible promoters induced with 1 mg/L doxycycline for 24 h.

### ChIP

Bio-ChIP was performed as described (Baubec et al. 2013).

### Immunoprecipitation and western blotting

Immunoprecipitation and western blotting was performed as described (Baubec et al. 2013; Domcke et al. 2015).

### Generation of *Dnmt1*, *Dnmt3a*, *Dnmt3b* knockout cell line

Deletions were generated from TC-1 ES cells as described (Domcke et al. 2015).

### Reporter assay data analysis

For additional description on this section, see Supplemental Methods.

#### Barcode to promoter assignment

FASTQ files were trimmed to the promoter sequence and aligned to mm9 using Bowtie (Langmead et al. 2009) for libraries where the design allowed efficient alignment (see Supplemental Table S2). For mutant promoter libraries, reads were matched to the reference sequences using the “stringdistmatrix” function in R (van der Loo 2014), which, unlike Bowtie, does not limit the number of allowed mismatches. This was necessary due to the high error rate toward the end of very long (2 × 300 nt) Illumina reads. We allowed a total of 20% errors in both reads (i.e., in 600 bp sequenced) and applied a cutoff on the minimum distance (i.e., the number of mismatches) to the next closest reference of 3 (Supplemental Fig. S1C).

Barcodes were extracted from each second read and matched to the aligned reads by read ID. Only barcodes that were associated with one unique sequence or with a sequence where the ratio of the second most abundant sequence to the most abundant sequence was below 0.3 were used for the analysis (Supplemental Fig. S1C).

#### Quantification of transcriptional activity

Transcriptional analysis was performed in triplicate following genomic insertion. Barcode sequences were extracted, and the frequency of each barcode sequence was calculated to get counts for each sample. Genomic DNA and RNA samples were scaled to each other by normalizing to the smaller total number of counts. Only barcodes that were sufficiently represented on genomic DNA (more than 20 reads after normalization) were used for further analysis. In case a barcode was sufficiently represented on genomic DNA but not sequenced in the RNA fraction, we assumed that this reflects lack of activity and assigned 0 counts to the RNA barcode. Enrichments of barcodes in the RNA sample were then calculated as *n*_r_/*n*_d_ + α, where *n*_r_ are the RNA counts and *n*_d_ the DNA counts for a particular barcode and α represents a pseudocount (for a derivation, see Supplemental Methods). The first ratio can be understood as being proportional to the RNA counts per single cell, to which a constant pseudocount of α is added. α was set to 0.05 as this was the smallest value of α that roughly stabilized the variance in all libraries. Log_2_ promoter activities were calculated as the mean of the log_2_ enrichments of all barcodes assigned to the particular promoter. All plots displaying expression data show mean log_2_ activities of three replicates unless indicated otherwise.

#### Significance calculations

The significance of Spearman's correlations was calculated using permutation tests.

### ChIP-seq data analysis

Samples were mapped to the mm9 assembly of the mouse genome using the R package QuasR (Gaidatzis et al. 2015), which internally uses Bowtie (Langmead et al. 2009). We do not expect changes to our conclusions if we used the more recent version of the mouse genome assembly, mm10, instead of mm9, as our analysis is focused on regions outside of repeats. These nonrepetitive regions were already well sequenced in mm9.

Peaks were called using Peakzilla with default parameters (Bardet et al. 2013). Position weight matrices of motifs were generated based on called peaks in the bio-ChIP-seq data using HOMER on all peaks with default parameters (Heinz et al. 2010). The motif score was defined as the commonly used log-odds score (in log_2_ scale) with respect to a uniform background. For more details, see Supplemental Methods.

### Precision-recall analysis

For a given cut-off on either CpG density (observed over expected), motif score, or the probability of being bound predicted by a logistic regression that uses both CpG density and motif score as input, the fraction of genomic windows larger or equal to the cut-off that are bound (precision) and the number of bound genomic windows larger or equal to the cut-off divided by the total number of bound windows (recall) were calculated. Precision-recall curves were determined by varying the corresponding cut-offs over the entire range of values (100 values from minimum to maximum in equally sized steps). For more details, see Supplemental Methods.

#### Methylation data processing

Sequences were aligned to mutant promoter libraries using QuasR (Gaidatzis et al. 2015) default settings for bisulfite-converted samples. DNA methylation was quantified using the QuasR function qMeth, and promoter methylation levels were calculated as the average methylation of all CpGs per promoter.

### Published data sets

The following ChIP-seq data sets were downloaded from GEO: NANOG, MYCN, POU5F1 (OCT4), SMAD1, SOX2, STAT3, TFCP2L1, ZFX (GSE11431) (Chen et al. 2008), REXO1 (GSE36417) (Gontan et al. 2012), TBX3 (GSE19219) (Han et al. 2010), TCF3 (GSE11724) (Marson et al. 2008), YY1 (GSE31786) (Vella et al. 2012), ZIC2 (GSE61188) (Luo et al. 2015), CTCF (GSE30206/GSM747534) (Stadler et al. 2011), NFYA (GSE25533/GSM632038) (Tiwari et al. 2011), NRF1 (GSE67867/GSM1891641) (Domcke et al. 2015), REST (GSE27148/GSM671093) (Arnold et al. 2013).

DNase hypersensitivity data set was retrieved from GEO under the accession number (GSE67867) (Domcke et al. 2015).

The CAGE data set was downloaded from the FANTOM Consortium homepage (http://fantom.gsc.riken.jp) (The FANTOM Consortium and the RIKEN PMI and CLST [DGT] 2014).

For a complete overview, see Supplemental Table S3.

## Data access

The raw sequencing data generated in this study have been submitted to the NCBI Gene Expression Omnibus (GEO; https://www.ncbi.nlm.nih.gov/geo/) under accession number GSE116704. Sequences and primers of expression libraries are provided in Supplemental Files.

## Supplementary Material

Supplemental Material
