# Supplementary material for: CG dinucleotides enhance promoter activity independent of DNA methylation
Source: Genome Res. 2019 Apr;29(4):554–63. doi: 10.1101/gr.241653.118 (PMC6442381; doi:10.1101/gr.241653.118)
Supplement: Supplemental Material [file supp_29_4_554__index.html]

CG dinucleotides enhance promoter activity independent of DNA methylation — Supplemental Material 

# CG dinucleotides enhance promoter activity independent of DNA methylation

## Supplemental Material

- Supplemental\_Fig\_S1.pdf
- Supplemental\_Fig\_S2.pdf
- Supplemental\_Fig\_S3.pdf
- Supplemental\_Fig\_S4.pdf
- Supplemental\_Fig\_S5.pdf
- Supplemental\_Table\_S1.xlsx
- Supplemental\_Table\_S2.xlsx
- Supplemental\_Table\_S3.xlsx
- Supplemental\_Methods.pdf
