# Supplementary material for: CG dinucleotides enhance promoter activity independent of DNA methylation
Source: Genome Res. 2019 Apr;29(4):554–63. doi: 10.1101/gr.241653.118 (PMC6442381; doi:10.1101/gr.241653.118)
Supplement: Supplemental Material [file supp_gr.241653.118_Supplemental_Fig_S1.pdf]

Supplemental Figure 1

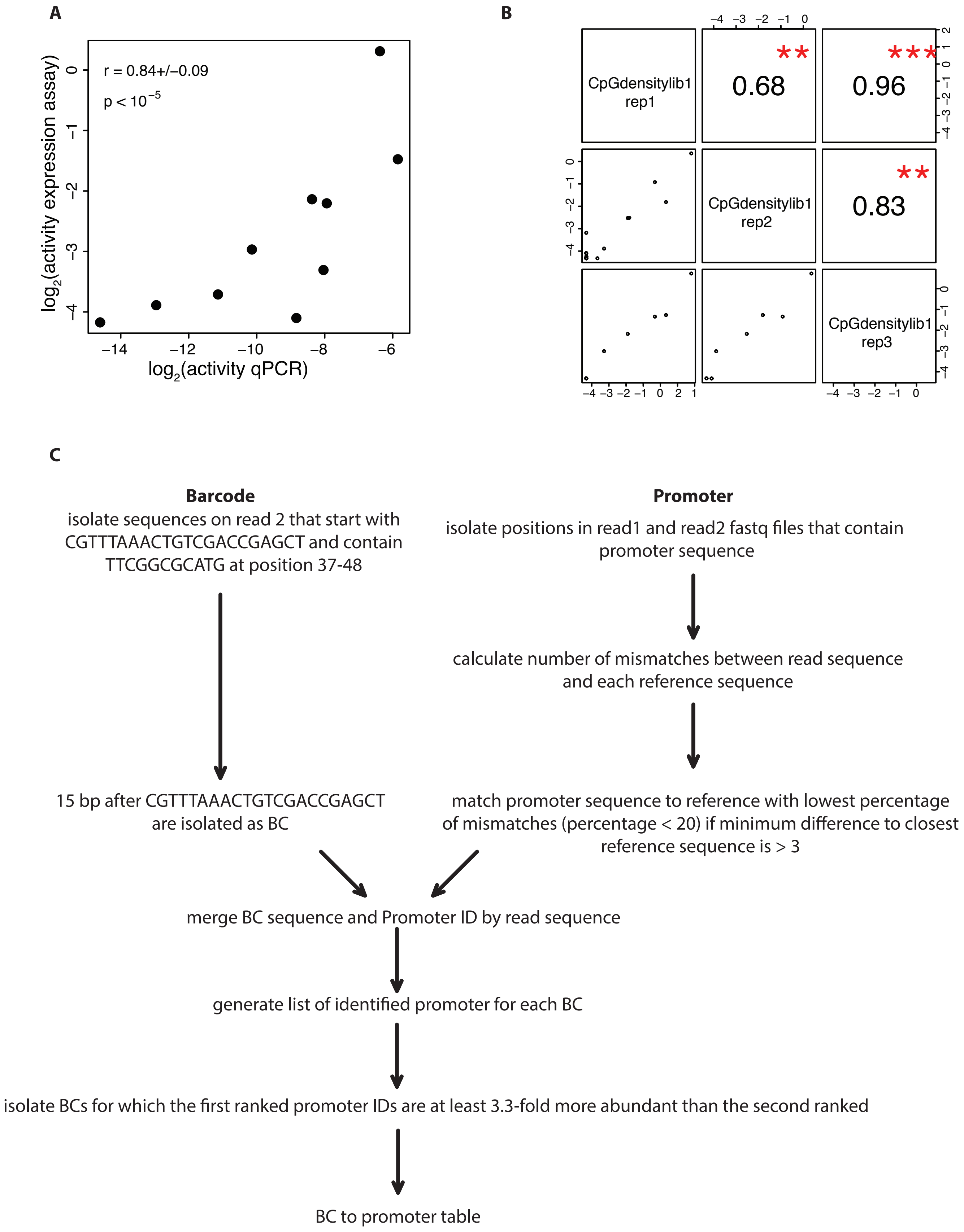

Supplemental Figure 1:

(A) Scatterplot displaying activity of promoter mutants measured by RT-qPCR in single clones with primers targeting the GFP transgene versus activity in the reporter assay. The average Spearman's correlation coefficient of all three replicates of the reporter assay (+- one standard deviation) and its significance is indicated in the upper left part of the scatterplot. P-values were determined based on an approximate permutation test (see Methods). The correlation coefficient indicates a good agreement between both measures.

(B) Pairwise scatterplot displaying Spearman's correlation of replicates of libraries used in Figure1. Stars indicate significance (≤0.05, ** ≤0.01, *** ≤0.001), calculated using an approximate permutation test (see Methods).*

(C) Workflow to match barcode and promoter sequences.
