# Supplementary material for: CG dinucleotides enhance promoter activity independent of DNA methylation
Source: Genome Res. 2019 Apr;29(4):554–63. doi: 10.1101/gr.241653.118 (PMC6442381; doi:10.1101/gr.241653.118)
Supplement: Supplemental Material [file supp_gr.241653.118_Supplemental_Fig_S2.pdf]

Supplemental Figure 2

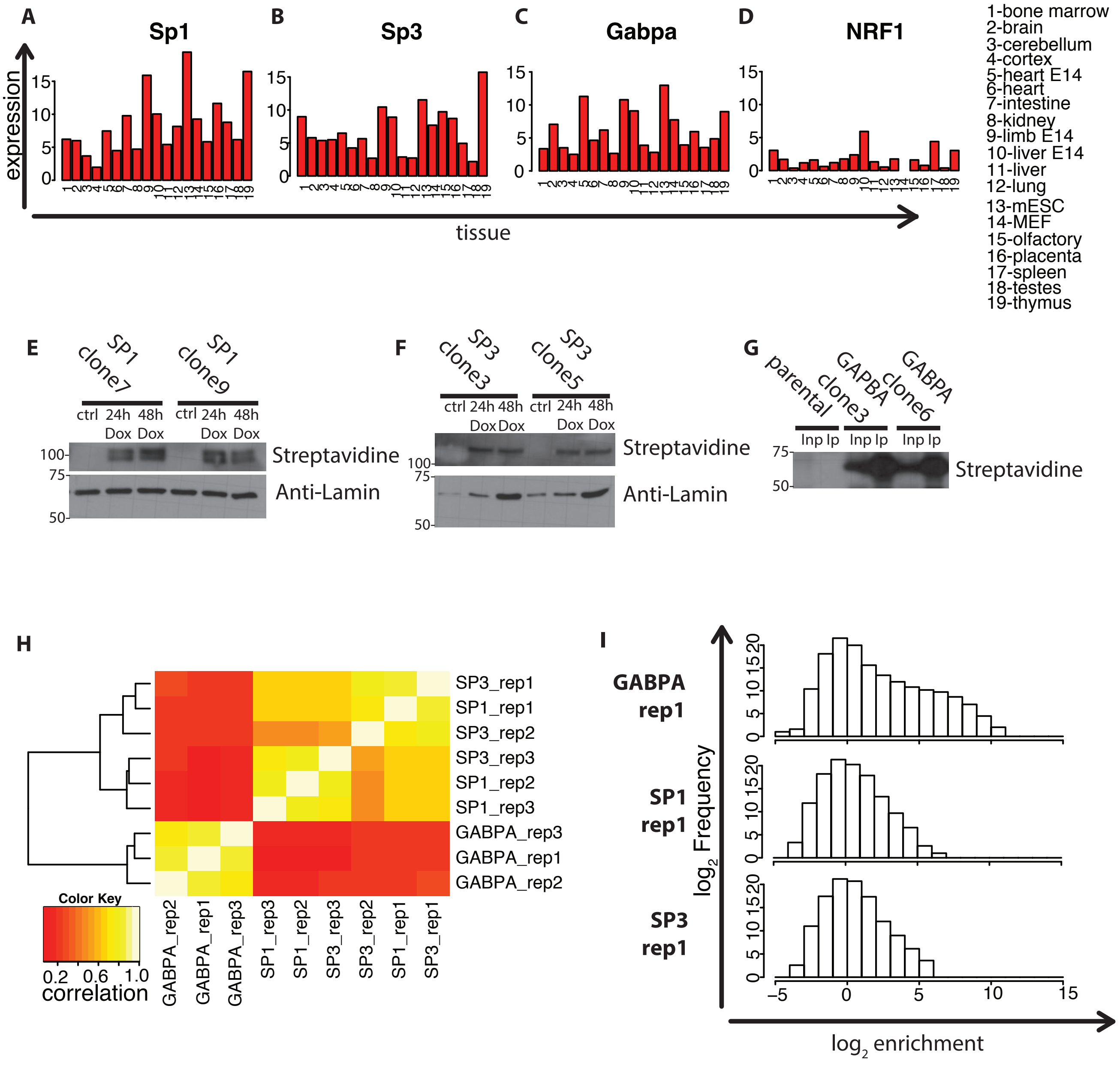

Supplemental Figure 2:

(A)-(D) SP1 (A), SP3 (B), GABPA (C) and NRF1 (D) are relatively broadly expressed. Expression levels (reads per kilobase per million) are shown as barplots across 19 different tissues. The tissues corresponding to the numbers are indicated on the right.

(E)-(F) Western blot using nuclear extracts of cells containing inducible Bio-SP1 (E) and Bio-SP3 (F) without induction (ctrl), 24h and 48h post induction with DOX. Biotinylated proteins were detected using streptavidin-HRP. For ChIP-seq experiments cells were induced for 24h with DOX.

(G) Western blot before (Input, 'Inp') and after immunoprecipitation (IP) with streptavidin beads. Proteins were isolated from the parental cell line without Bio-GABPA and from two clones (GABPA clone3 and GABPA clone7) that stably express Bio-GABPA under the control of a CAG-promoter.

(H) Heatmap of correlations of log2 enrichments over input in the union set of called peaks across all samples.

(I) Histogram of enrichments of GABPA, SP1 and SP3 ChIPs. One representative ChIP-seq replicate is displayed. Frequencies are shown in log2 scale.
