# Supplementary material for: CG dinucleotides enhance promoter activity independent of DNA methylation
Source: Genome Res. 2019 Apr;29(4):554–63. doi: 10.1101/gr.241653.118 (PMC6442381; doi:10.1101/gr.241653.118)
Supplement: Supplemental Material [file supp_gr.241653.118_Supplemental_Fig_S3.pdf]

Supplemental Figure 3

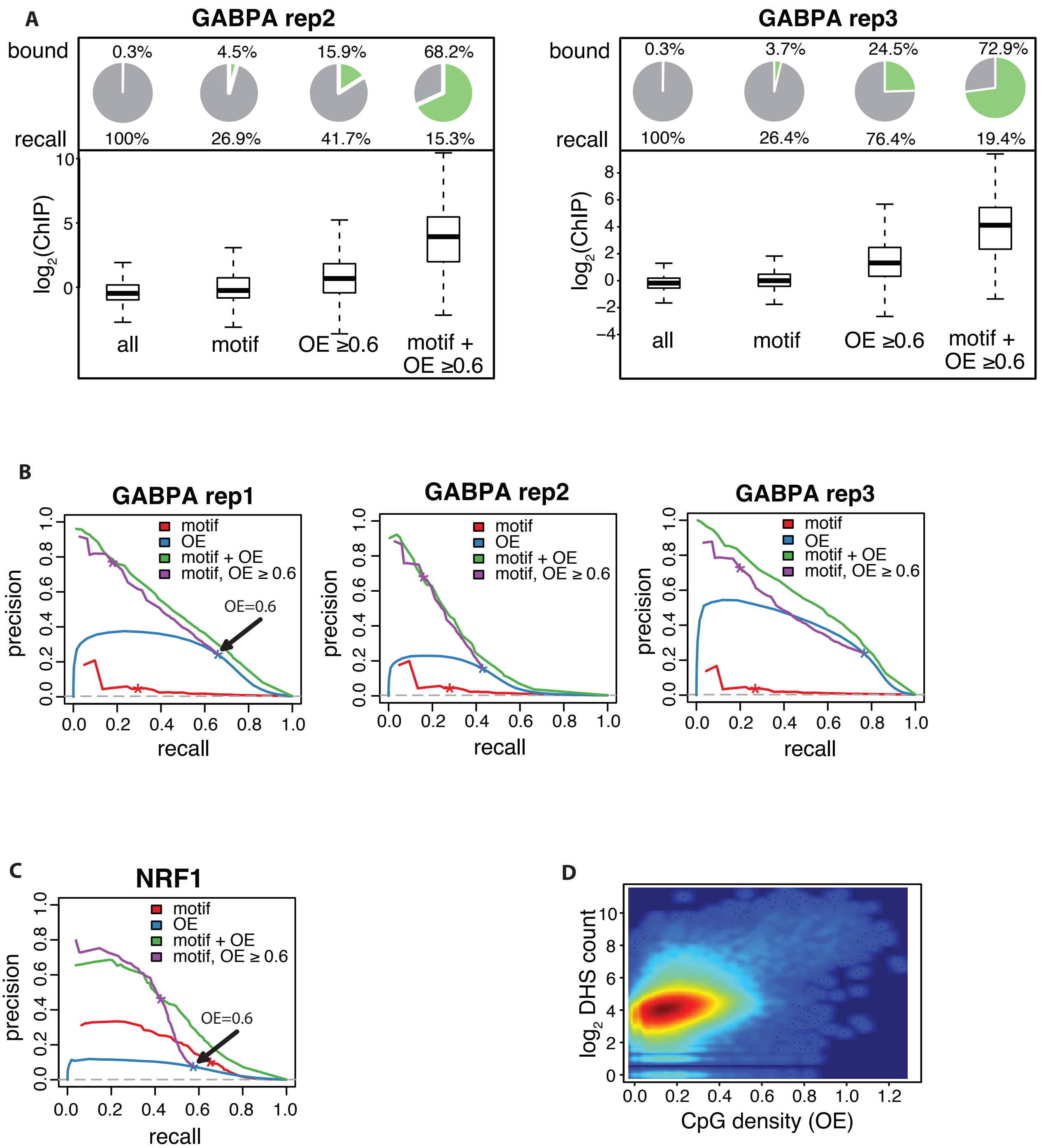

Supplemental Figure 3:

(A) Pie charts show, for different subsets, the percentage of GABPA-bound genomic windows (600 nt tiling windows, log2 enrichment over input > 2.5), with the percentage indicated above the pie chart ('bound'). 'recall' indicates the percentage of all bound genomic windows that are part of each subset. Corresponding boxplots of log2 ChIP enrichments are shown below the pie charts. 'all' stands for all windows, 'motif' for windows containing a motif that has a log-odds score ≥ 12 (log2 scale), 'OE ≥0.6' for windows with an OE ≥ 0.6 and 'motif + OE ≥ 0.6' for windows with both a motif with a log-odds score ≥ 12 and an OE ≥ 0.6. Results for the second (left) and third (right) replicate of GABPA are shown.

(B) Performance of different models for the prediction of the binding of GABPA based on motif score (motif) and/or normalized CpG density (see Methods). Precision-recall curves for the three replicates of GABPA are shown for increasing motif log-odds scores (from right to left, red) or increasing normalized CpG densities (OE, from right to left, blue). The performance of a logistic regression model that takes into account normalized CpG density (OE) and motif score is shown in green (Methods). The precision-recall curve for increasing motif scores restricted to CpG-rich regions ($OE \geq 0.6$) is shown in purple. The dashed grey line indicates the fraction of bound windows in the genome. The minimum normalized CpG density used to define CGIs is indicated by arrows and precision/recall values used for (A) and Fig 2D are marked by asterisks.
(C) as in (B) but for NRF1. Asterisks indicate the precision/recall values used in Fig 2C.
(D) Normalized CpG density correlates with accessibility. Scatterplot show normalized CpG density versus accessibility as measured by DNAse hypersensitivity (data from Domcke et al. 2015).
