# Supplementary material for: CG dinucleotides enhance promoter activity independent of DNA methylation
Source: Genome Res. 2019 Apr;29(4):554–63. doi: 10.1101/gr.241653.118 (PMC6442381; doi:10.1101/gr.241653.118)
Supplement: Supplemental Material [file supp_gr.241653.118_Supplemental_Fig_S4.pdf]

Supplemental Figure 4

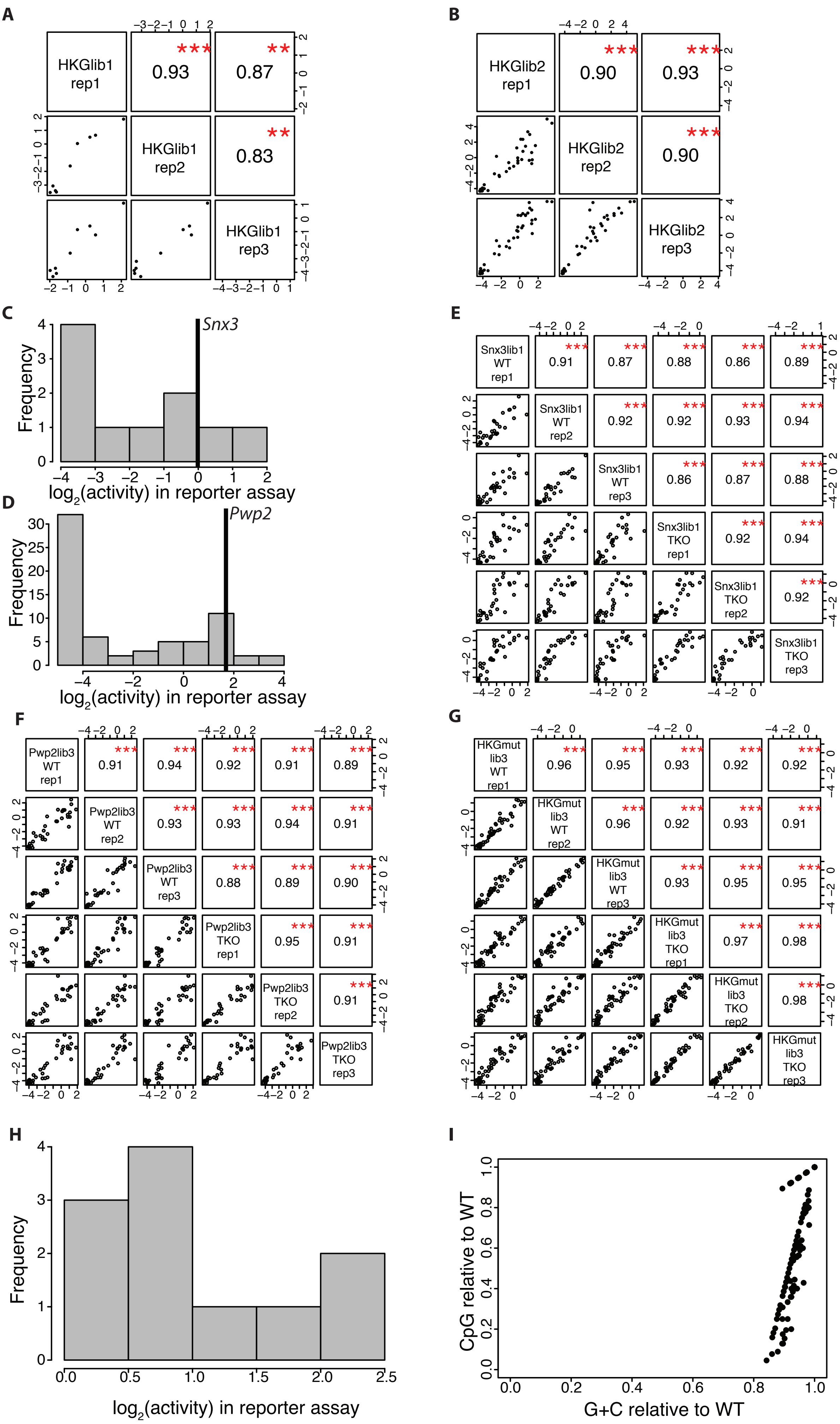

Supplemental Figure 4:
(A-B) Pairwise scatterplot of replicates of two libraries containing CGI promoters to identify candidates for mutation analysis. Correlations and their significance as in Supplemental Figure 1B.
(C)-(D) Histograms of activities of two libraries containing CGIs. Vertical lines indicate the activity of Snx3 (C) and Pwp2 (D).
(E) Pairwise scatterplot of replicates of Snx3 promoter CpG mutants (cf Fig 3B). Correlations and their significance as in A).
(F) Pairwise scatterplot of replicates of Pwp2 promoter CpG and TF motif mutants (cf Fig 3C). Correlations and their significance as in (A).
(G) Pairwise scatterplot of replicates of the library with CpG mutants of 11 different promoters (cf Fig 3D). Correlations and their significance as in (A)
(H) Histogram of WT activities of the 11 tested promoters (cf Fig 3D).
(I) Scatterplot of G+C and CpG densities of CpG mutants relative to the corresponding WT sequences.
