# Supplementary material for: CG dinucleotides enhance promoter activity independent of DNA methylation
Source: Genome Res. 2019 Apr;29(4):554–63. doi: 10.1101/gr.241653.118 (PMC6442381; doi:10.1101/gr.241653.118)
Supplement: Supplemental Material [file supp_gr.241653.118_Supplemental_Fig_S5.pdf]

Supplemental Figure 5

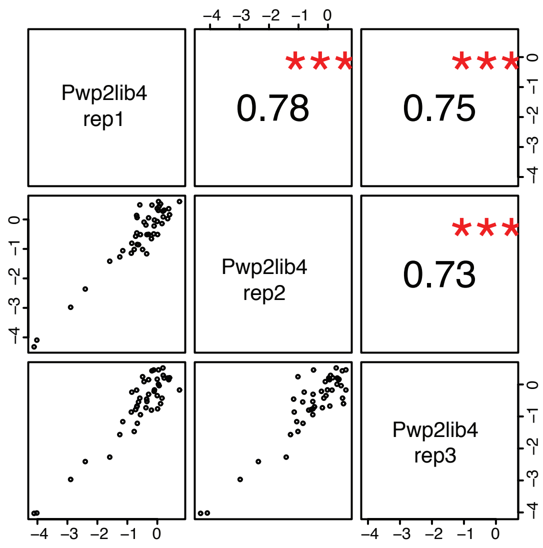

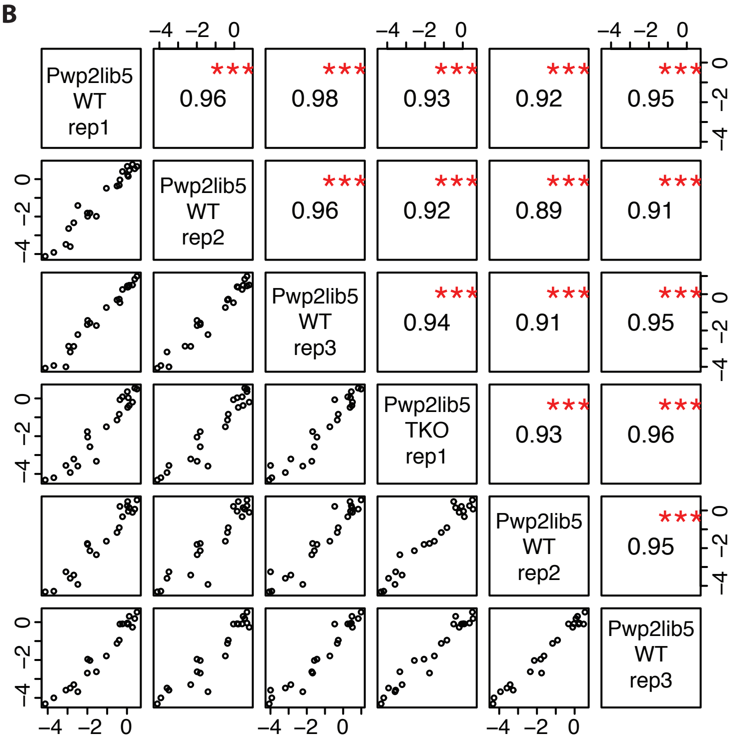

C

	Dnmt1	*Dnmt3a*	*Dnmt3b*
Allele 1	4bp del	2bp del	4bp del
Allele 2	5bp del	2bp del	4bp del

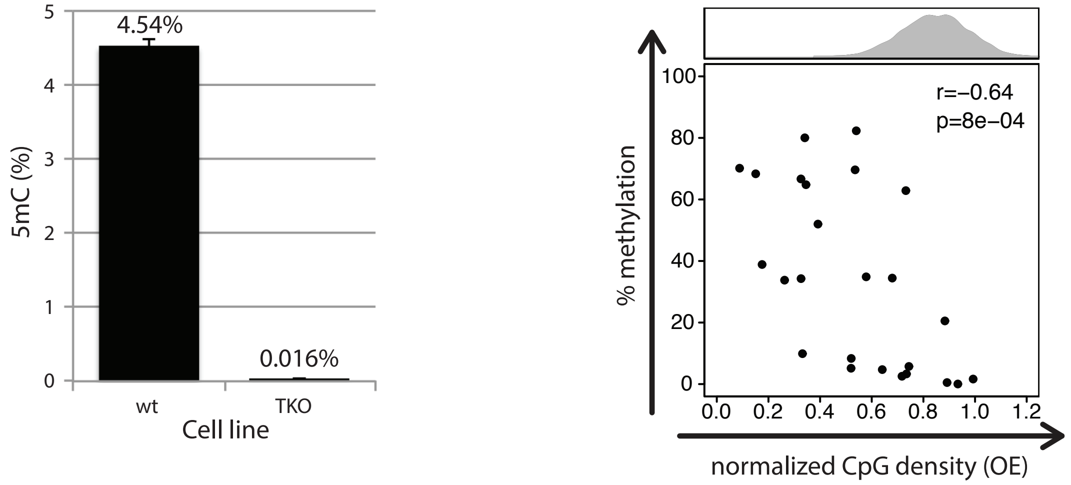

Supplemental Figure 5:
(A) Pairwise scatterplot of replicates of the library containing Pwp2 10bp window mutants (cf Fig 4B). Correlations and their significance as in Supplemental Figure 1B.
(B) Pairwise scatterplot displaying correlations of replicates of library containing constructs with an artificial sequence context (cf Fig 5C and D). Correlations and their significance as in Supplemental Figure 1B.
(C) Table of number of deleted bases in the Dnmt1, Dnmt3a, Dnmt3b knock-out cell line (TKO).
(D) Barplot of Mass-Spectrometry quantification of DNA methylation levels in the WT and TKO cell lines.
(E) Scatterplot displaying normalized CpG density versus DNA methylation at CpG mutant promoters. Spearman's correlation and p-value of an approximate permutation test are indicated (see Methods).
