# Supplementary material for: CG dinucleotides enhance promoter activity independent of DNA methylation
Source: Genome Res. 2019 Apr;29(4):554–63. doi: 10.1101/gr.241653.118 (PMC6442381; doi:10.1101/gr.241653.118)
Supplement: Supplemental Material [file supp_gr.241653.118_Supplemental_Methods.pdf]

Supplemental Methods:

Cell culture

Mouse ES cells were cultured on plates coated with 0.2% gelatine in DMEM medium with 15% fetal calf serum, 2mM L-glutamine, 1 x non-essential amino acids, LIF and 0.001% β-mercaptoethanol. Cells were incubated at 37 °C with 7% CO_2. TC-1 cells with a RMCE site in the *beta-globin* locus were used for integration of expression libraries for the transcriptional reporter assay (Lienert et al. 2011). *Dnmt1*, *Dnmt3a* and *Dnmt3b* were deleted in these cells using CRISPR-Cas9 gene editing as previously described (Domcke et al. 2015). Mouse HA36 ES cells (mixed 129-C57Bl/6 strain) were used for Rambio (Baubec et al. 2013).

Reporter Assay Data Analysis

Quantification of transcriptional activity

Barcodes correspond to the reverse complement of the sequence between "CGTTTAAACTGTCGACCGAGCT" and 'TTCGGCGCATG" on the second read. To determine the pseudo-count α used in the calculation of barcode enrichments, mean-variance relationships for $\log_2$ enrichments per barcode of the three replicates of each library were inspected for the following values of α: 0.001, 0.01, 0.05. 0.1. Finally, α was set to 0.05 as this was the smallest value of α that roughly stabilized the variance in all libraries. This value of α sets the lowest measurable enrichment level to 1/20, which is intuitive since the noise is dominated by the enrichment of the barcodes with lowest DNA read counts (which is defined to be > 20).

Only Promoters that were covered in at least 2 out of 3 replicates with at least three different barcodes were used for downstream analysis, except for Pwp2lib3 where this threshold was decreased to 1. All plots displaying expression data in this study show mean $\log_2$ activities of three replicates unless indicated otherwise. For HKGlib3, Pwp2lib3, Pwp2lib5 and Snx3Lib1, activities were calculated relative to the WT construct (as the ratio of the activity of a given construct and the activity of the WT construct) before $\log_2$-transforming and averaging over replicates.

Statistical model for the estimation of barcode enrichments

If we denote the (unknown) expression of a given construct in a given cell c as e_c and if we assume for the moment that we would know the (scaled) RNA barcode counts for a single cell n_c, then we can write the probability conditional probability of these counts n_c as $P(D|e_1,..,e_m) = \prod_c (\frac{e_c}{\sum_{c'} e_{c'}})^{n_c}$, where m is the total number of cells and where we have assumed that the probability of getting a read from a particular barcode is proportional to the expression of the reporter gene of that particular construct. If we assume a symmetric Dirichlet prior for each of the probabilities $P(e_1,..,e_m) \propto \prod_c (\frac{e_c}{\sum_{c'} e_{c'}})^{\beta-1}$, then we can write the posterior probability of the expression levels given the data as $P(e_1,..,e_m|D) \propto \prod_c (\frac{e_c}{\sum_{c'} e_{c'}})^{n_c+\beta-1}$. If we assume that each cell with the same barcode (and thus construct) has the same expression, we can simplify this expression as follows $P(e_1,..,e_k|D) \propto \prod_b \left(\frac{e_b}{\sum_{b'} m\lambda_{b'} e_{b'}}\right)^{n_b+m(\beta-1)}$, where k is the number of different barcodes, the product now runs over all barcodes, λ_b is the fraction of cells with barcode b in the population of cells, $m\lambda_b$ thus the absolute number of cells with barcode b and n_b (for notational simplicity) now denotes the total RNA counts of barcode b (which is what we measure). Differentiating the logarithm of this expression with respect to any of the e_i and setting the resulting expressions to zero, results in the following equation $\frac{e_1}{\frac{n_1}{\lambda_1}+m(\beta-1)} = \frac{e_2}{\frac{n_2}{\lambda_2}+m(\beta-1)} = \cdots = \frac{e_k}{\frac{n_k}{\lambda_k}+m(\beta-1)} = const$ or equivalently $e_i \propto \frac{n_i}{C\lambda_i} + \alpha$, where we define the new constant α as α:=Cm(β-1) and C is a proportionality constant such that $C\lambda_i$ equals the (scaled) DNA counts for barcode b.

Significance calculations

The significance of Spearman's correlations was calculated using permutation tests. In the case of measures that did not represent the average over several replicates, this was done using the function *spearman_test* (with argument distribution=approximate (B=100000)) as implemented in the R package *coin* (Hothorn et al. 2008). The number of randomly sampled permutations of the data were set to 100'000 and a two-sided test was used.

In cases where one of the two measures corresponded to the average over three replicates (y1, y2 and y3, in all cases corresponding to measured activities in three replicates) and the other measure to a single replicate (x, CpG density observed/expected

or luciferase activity), the significance of the Spearman's correlation was calculated by repeating the following 100'000 times: y1, y2 and y3 were independently randomly permuted and the average of the Spearman's correlations of y1 and x, y2 and x and y3 and x was calculated. In the same way, the average correlation between the ys and x was determined for the measured, unpermuted data. The p-value was then determined as the fraction of times the absolute value of the average correlation of the measured data was at least as large or larger that the absolute value of the average correlation in the permuted data (two-sided test).

To determine the significance of the correlation between the difference in average $\log_2$ activity in TKO and WT and CpG density observed/expected, we calculated the very same correlation for all possible 19 permutations of the three WT and three TKO labels among the six activity measurements and determined the p-value as the fraction of times the true labelling resulted in a correlation that was at least as large or larger than the correlation resulting from a permutation of the labels. Also here, the absolute values of the Spearman's correlation were used, thus resulting in a two-sided (exact) permutation test.

In the case of the correlation between promoter methylation and OE, where different numbers of replicate measurements were available for each promoter (44% with 1 measurement, 32% with 2 replicate measurements and the remaining 24% promoters with more than 2 replicate measurements), the vector of all methylation levels (including replicate measurements) were randomly permuted 100'000 times, aggregated to average promoter levels and, for each permutation, the absolute value of the Spearman's correlation of average promoter levels with OE was calculated. The p-value was then determined as the fraction of times the absolute value of the true correlation was at least as large or larger than the correlations resulting from the permutations (two-sided test).

ChIP-seq data analysis

Counts for the union of all peaks were calculated using the QuasR function qCount (Gaidatzis et al. 2015) with reads shifted by 80nts based on the distance of average profiles of reads mapping in the same or opposite orientation of high scoring motifs. Enrichments over input were calculated after normalizing counts to the mean library size and adding a pseudo-count of 8. For all other plots 'bound' was defined as an enrichment larger than 2.5-fold over input in $\log_2$ space.

Precision-recall analysis

For the model that allows predictions based on motif score only within CGIs (defined by OE >= 0.6, purple curve in Supplemental Figures 3B and C), the motif score outside of CGIs was artificially set to a value lower than the smallest motif score within CGIs and only cut-offs larger than this value were used to determine the precision-recall curve. For the logistic regression, motif score and OE were binned as their relationship to the ChIP enrichments was not clearly linear. The motif score was binned into one bin for all windows without a motif match (motif score < 5), and 9 bins covering equally sized intervals over the entire range of motif scores. Analogously, OE was binned into 10 bins covering equally sized intervals from 0 to 1.1 where any values larger than 1.1 (only 360) were assigned to the top bin. The logistic regression was trained on all windows on chromosome 1 and used to predict the probability of being bound on all the remaining chromosomes. All precision-recall curves were determined using the windows on all chromosomes except chromosome one.

References:

Baubec T, Ivánek R, Lienert F, Schübeler D. 2013. Methylation-Dependent and -Independent Genomic Targeting Principles of the MBD Protein Family. *Cell* **153**: 480–92.

Domcke S, Bardet AF, Ginno PA, Hartl D, Burger L, Schübeler D. 2015. Competition between DNA methylation and transcription factors determines binding of NRF1. *Nature* **528**: 575–579.

Gaidatzis D, Lerch A, Hahne F, Stadler MB. 2015. QuasR: Quantification and annotation of short reads in R. *Bioinformatics* **31**: 1130–1132.

Hothorn T, Hornik K, van de Wiel MA, Zeileis A. 2008. Implementing a Class of Permutation Tests: The coin Package. *J Stat Softw* **28**: 1–23.

Lienert F, Wirbelauer C, Som I, Dean A, Mohn F, Schübeler D. 2011. Identification of genetic elements that autonomously determine DNA methylation states. *Nat Genet* **43**: 1091–7.
